# Metallothioneins in Failure of Dental Implants and Periodontitis Down Syndrome Patients

**DOI:** 10.3390/genes10090711

**Published:** 2019-09-14

**Authors:** Maria Baus-Domínguez, Raquel Gómez-Díaz, Jose-Ramón Corcuera-Flores, Daniel Torres-Lagares, José-Cruz Ruiz-Villandiego, Guillermo Machuca-Portillo, José-Luis Gutiérrez-Pérez, María-Angeles Serrera-Figallo

**Affiliations:** 1 Oral Surgery Department, Dentistry Faculty, University of Seville, 41009 Seville, Spain; mbaus95@gmail.com; 2 Instituto de Biomedicina de Sevilla, 41007 Seville, Spain; rgomez-ibi@us.es; 3 Dentistry in Handicapped Patients Department, Dentistry Faculty, University of Seville, 41009 Seville, Spain; joserra_64@hotmail.com (J.-R.C.-F.); gmachuca@us.es (G.M.-P.); maserrera@us.es (M.-A.S.-F.); 4 Dentistry in Handicapped Patients Department, Quirón Hospital, 20012 San Sebastián, Spain; maserrera@ono.com; 5 Oral and Maxillofacial Unit, Virgen del Rocio Hospital, 41009 Seville, Spain

**Keywords:** bone biology, clinical outcomes, gene expression, systemic health, systemic disease, osseointegration, periodontal disease, Down syndrome

## Abstract

Background: Sometimes dental implants seem to be the only therapeutic alternative for the oral rehabilitation of patients with Down syndrome, given that they usually lose all their teeth early due to suffering aggressive periodontitis and they do not usually have the skills required to wear removable prostheses. However, the evolution of dental implants in these patients shows very adverse results. It is possible that basal genetic alterations, or at least some characteristics of these, may underlie these clinical results. The metabolic pathway of metallothioneins, molecules with an important influence on bone metabolism, could be one of the said alterations. Aims: To determine whether the expression of metallothioneins (MTs) and their metabolic pathway may be identified and related to the periodontitis and lack of osseointegration of dental implants in Down syndrome patients. Materials and Methods: Retrospective study of cases and controls by comparing patients with Down syndrome, periodontal disease, and implant failure (four patients, test group) with patients with Down syndrome, without periodontal disease, and without implant failure after two years of following (seven patients, control group), by extracting peripheral blood at the time of the dental examination to extract RNA and its subsequent processing in relation to gene expression of the metabolic pathway of metallothioneins. Results: The results identified low expression in the group of patients with periodontal disease and implant failure of genes MT1E, MT1H, MT1X, MT1A, MT1B, MT1C, MT1L, MT2A, MT1M, and MT1G. Conclusions: The low MT1 and MT2 gene expression seems to be related to the onset of periodontal disease and implant rejection in Down syndrome patients, although more data are required to confirm whether this relationship is due to one of the two conditions, to both independently, or to the two jointly—this last option being indicated by our current study.

## 1. Introduction

Down syndrome (DS) covers a large number of pathologies that affect practically every system in the body, including the cardiovascular, hematological, skeletal muscle, nervous, endocrine, and digestive systems. It affects patients’ oral health to a large extent and, accordingly, determines their dental treatment.

Despite the enormous number of orofacial and dental manifestations that can be found in Down syndrome patients, the main problem that this study concentrates on arises when these patients need dental replacements or complete rehabilitation due to the loss of their teeth, or when these have never existed (dental agenesis).

Given the intellectual disability of these patients, removable prostheses are often not advisable due to the difficulty in handling them, putting them in, or looking after them [1]. Fixed dental prostheses may be a better therapeutic option for the treatment of these patients, especially those with moderate or profound intellectual disability, since they tend to be well tolerated [2], although it is important to highlight that they must be well maintained (cleaned). For this reason, treatment with implants is sometimes the only option for supporting fixed dental prostheses in this type of patient [3,4].

In this context, there are a few case studies and controls, such as the one by Corcuera-Flores et al. [5], which show how the placing of implants in these patients may have adverse results. A large number of DS patients have lost teeth through aggressive periodontitis; could it be possible that some similar destructive process of the peri-implant support is taking place? Certain syndromes related to implant failure or with specific microbiological characteristics have been described [6,7,8,9].

In this connection, it would be interesting to know whether the metabolic pathway of metallothioneins is altered and could be the cause of the failure of implant treatment and/or periodontal disease in Down syndrome patients, which could be explained by an implication of these in the osseointegration process and/or in the aggressive periodontitis that DS patients present.

Metallothioneins (MTs) are a family of small metal proteins rich in cysteine, extremely different, generally of low molecular weight, which are capable of bonding to heavy metals both physiological (Zn and Cu) and xenobiotic (Cd, Hg, and Ag), via the thiol groups (‒SH) of their cysteine residues. They are involved in the homeostasis and transport of essential metals—more specifically, in the protection of cells against the toxicity of heavy metals [10,11]. In humans, four genes that codify these molecules grouped in tandem (MT1 to MT4) are known. These genes are oriented cent-MT4-MT3-MT2-MT1-tel and flanked by BBS2 and NUP93 on chromosome 16. Furthermore, MT1 presents 13 grouped duplicates, isoforms (MT1A to MT1J, MT1L, MT1M, and MT1X), five of which (MT1L, MT1J, MT1D, MT1C, and MT1L) are predicted not to be active forms, and pseudogenes [12]. Currently it is thought that pseudogenes can act as possible regulators of the genes they come from and are not junk DNA, as had been thought up to now.

In tissues with a high rate of cell proliferation, a high rate of metallothionein synthesis has been detected. When these bind to zinc or copper, they act as a reservoir for the synthesis of apoenzymes. In zinc it is an important cofactor and, therefore, essential for cell growth. It has catalytic functions; more than 3000 proteins contain it in their structures; it modulates cell signaling, and kinase and phosphatase protein activities, as well as transcription factors; it also modulates GMPc metabolism, kinase C and MAPK activities, and MTF-1 activity, among many others [12,13].

The study is based on this question, with the following null hypothesis: the metabolic pathway of metallothioneins is expressed in the same way in the following groups of patients:

- Down syndrome patients who have suffered aggressive periodontitis and for whom implant treatment has failed.
- Down syndrome patients who have not suffered aggressive periodontitis and have a positive experience of implant treatment (more than two years’ follow-up without bone loss problems (grade 1 on the Lavergall and Jansson scale) [14].

The alternative hypothesis is that the metabolic pathway of metallothioneins is expressed inconsistently, either upregulated or downregulated, among the two groups of patients studied.

## 2. Experimental Section

### 2.1. Type of Study

Retrospective case study and controls approved by the corresponding Ethics Committee (Hospital Virgen del Rocío Ethical Committee—Case file PI-0081-2016). The study is descriptive and observational, where the only invasive procedure performed on patients is the collection of a small amount of blood and a dental examination. The patient (or the person responsible for them) gives their consent based on the direct benefits to the research patient.

### 2.2. Selection of Patients and Study Groups

The study groups were:

(1) Down syndrome patients without periodontal disease and successful implants after two years (-PD-FI), (2) Down syndrome patients with periodontal disease and failed implants after two years (+PD+FI).

A failed implant is understood as lost after two years’ follow-up, or peri-implant bone loss of at least grade 2 on the Lagervall and Jansson scale [14].

It was intended to include 10 patients in each of these groups, for a total of 20 patients studied. In actual practice, the number of subjects in the study was reduced, with a total of seven patients for group 1 and four patients for group 2.

The exclusion criteria were as follows:

- Non-Down syndrome patients
- Patients receiving treatment that could possibly affect bone metabolism, such as long-term corticosteroids, bisphosphonates, or monoclonal antibodies.
- Patients treated with short implants, or with immediate loading implants.
- Patients with active or untreated periodontal disease.
- Patients with implants inserted less than two years ago.

Once the patients had been identified, and the existence or not of periodontal disease and its grade had been confirmed from the medical records, patients were included in one of the two approaches (with periodontal disease and negative evolution implants; without periodontal disease and positive evolution implants).

In patients with implants inserted, panoramic X-rays were taken for all patients who still had implants to calculate the marginal bone loss (MBL). The panoramic X-rays were assessed to calculate the MBL of the bone loss using the Lagervall and Jansson index [14] and validated for its use in this type of study by Corcuera-Flores et al. [15]. This method divides the implants into four groups according to their MBL:

- Grade 0: implants with no marginal bone loss.
- Grade 1: marginal bone loss of one-third or less of the total length of the implant.
- Grade 2: one-third, but less than two-thirds, of the total length of the implant.
- Grade 3: marginal bone loss greater than two-thirds of the total length of the implant.
- A fifth group (Grade 4) was added of those patients where the implant was lost.

The panoramic X-rays taken at the time of inserting the implant were reviewed to check if any bone defect around it had occurred in the postoperative period, and that no defect was caused during its insertion. Subsequently, X-rays were taken after two years’ evolution (or at the nearest possible time point, but always greater than two years).

### 2.3. Sampling and Total RNA Isolation

At the time of examining the patients selected and included, sampling was undertaken, which comprised two collections of blood per patient collected in PAXgene tubes, reference 762165 (100 tubes), with the ultimate aim of RNA collection.

The transfer of samples to the processing center is under refrigeration (2–8 °C) for up to five days. For longer storage periods, the tubes are kept at −20 °C or −80 °C.

RNA sample collection was made using Qiagen’s PAXgene Blood (QIAGEN GmbH, Hilden, Germany) miRNA Kit (50 tubes) reference 763134. The collection was carried out at the Qiagen QIAcube (QIAGEN GmbH, Hilden, Germany) automated station.

Subsequently, a database of the samples was compiled wherein we specified, among other information, data relating to DNA and RNA quantification.

### 2.4. Gene Expression

Firstly, RNA concentrations were quantified using a Thermo Scientific™ NanoDrop 2000c visible light spectrophotometer (Santa Clara, CA, USA), in order to ensure correct processing of these before their conservation.

Secondly, a much more precise measurement was taken, using Thermo Scientific’s Qubit 3.0 fluorometer kit; in this case, only the samples selected for the first study of gene expression were added to the database.

The last step was a gene expression study to determine whether metallothioneins have differences in their expression in the different study groups.

The platform chosen for this study was Thermo Scientific’s GeneChip^®^ Scanner 3000, and the chips chosen were Clariom S solutions for humans, mice, and rats, with more than 20,000 genes entered for measuring expression levels.

The RNA chosen was amplified and hybridized using Thermo Scientific’s GeneChip^®^ WT PLUS Reagent Kit.

**Figure 1:**
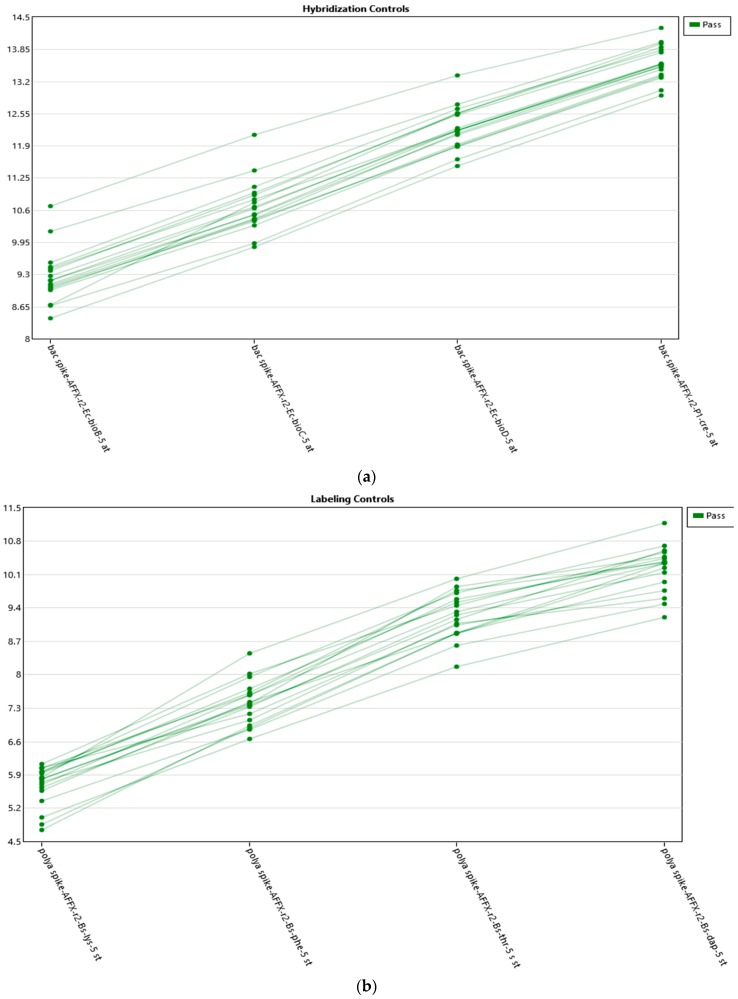
(**a**) Hybridization controls. Graphs on the quality controls provided by the platform, indicating that the experiment has been conducted correctly. (**b**) Labeling controls. Graphs on the quality controls provided by the platform, indicating that the experiment has been conducted correctly.

**Figure 2:**
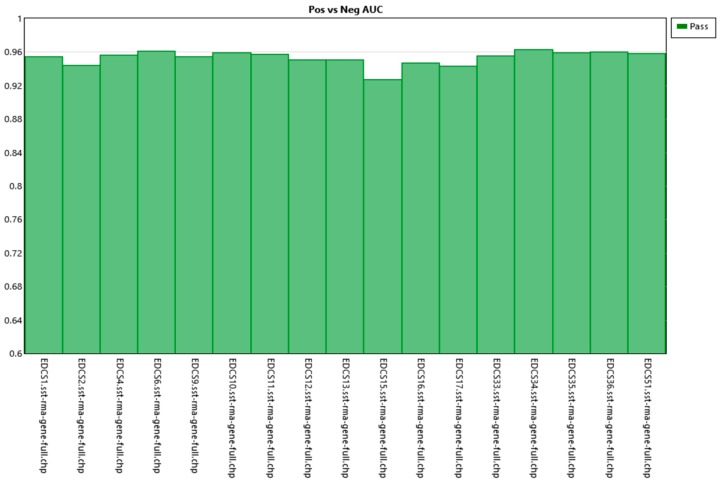
Positive vs. negative AUC. Shows the value of the samples analyzed. It can be seen that all are close to a value of 1, which shows that the samples behave correctly and homogeneously.

Amplification was performed from an initial total of 55 grams and was undertaken in accordance with the manuals described in the GeneChip^®^ WT PLUS Reagent Kit. Scanning with Thermo Scientific’s GeneChip^®^ Scanner 3000 was performed following the protocols for loading of the array cartridges (Figure 1 and Figure 2).

Finally, analysis was performed normalizing and using the robust multi-array (RMA) method, and the analysis of the different gene expression was undertaken using transcriptome analysis console (TAC, Affymetrix) software.

## 3. Results

### 3.1. Participants

The patients included in the study are classified into different groups according to their diagnosis of periodontal disease and the evolution of their implants over two years.

**Figure 3:**
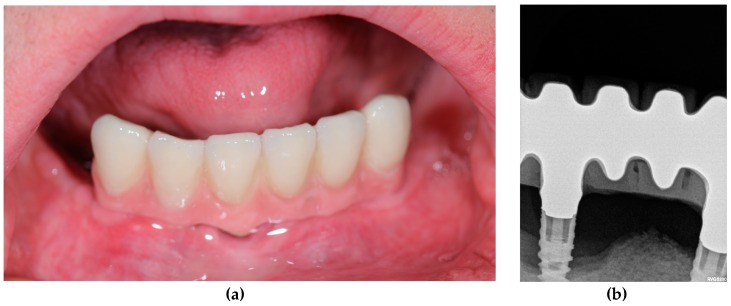
Patient 2 (+PD+FI). (**a**) Clinical view. (**b**) Radiographical view. After the loss of all teeth due to severe periodontitis, four implants were placed, two of which are affected by peri-implantitis with severe bone loss at the end of the follow-up.

**Figure 4:**
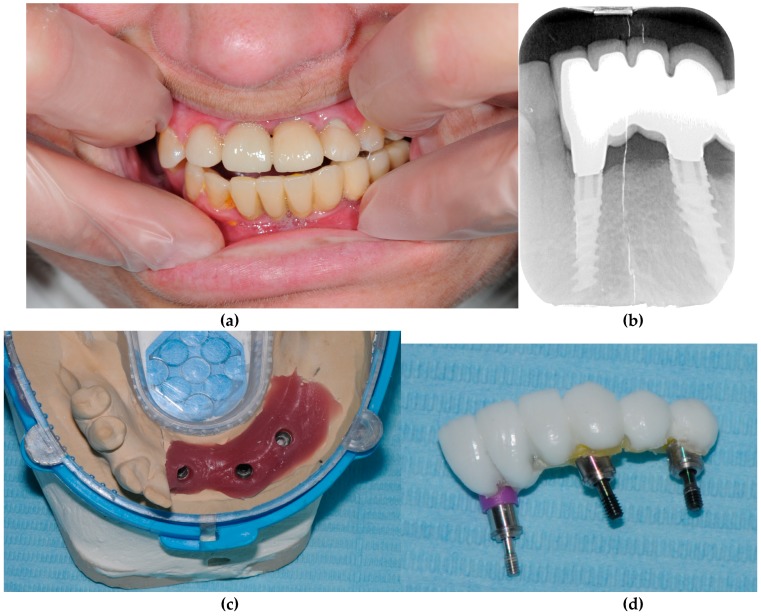
Patient 7 (-PD-FI). (**a**) Clinical view. (**b**) Radiographical view. (**c**) Prosthetic work model. (**d**) Dental prosthesis before being placed. The three implants were still in good shape two years after their placement.

A total of four patients with Down syndrome with periodontal disease and failed implants (+PD+FI) were compared with seven Down syndrome patients without periodontal disease and without failed implants (positive evolution of implants) (-PD-FI) (Table 1, Figure 3 and Figure 4).

The only reason why more patients were not included in the study was because the inclusion and exclusion criteria were strict and many of them were outside the designed sample. In the first instance, it was intended to include one more study group, Down S-syndrome patients w without periodontal disease but with failed implants. However, no patients with these characteristics were found. This could also be considered a result.

Demographic and clinical variables are taken from patients’ medical records.

### 3.2. RNA Analysis

The results obtained via RNA analysis using the Affymetrix Microarrays program, of the blood samples collected during the examination process, for the metallothioneins in the patients tested are shown in Table 2.

The ones obtained via RNA analysis using the Affymetrix Microarrays program appear to show a low gene expression for metallothioneins, with a statistically significant value (*p* < 0.05).

The low gene expression of MTs, in particular, of isoforms of MT1E (Fold change (FD) –2.71; *p* = 0.0014), MT1H (FD –2.39; *p* = 0.0018), MT1X (FD –3.09; *p* = 0.0021), MT1A (FD –2.82; *p* = 0.0023), MT1B and C (FD –2.95; *p* = 0.0024), MT1L (FD) –2.35; *p* = 0.0048), MT2A (FD) –2.35; *p* = 0.0072), MT1M (FD –2.37; *p* = 0.0092), MT1G (FD –2.24; *p* = 0.0118), may be an indicator that the metallothionein pathway is altered in certain patients who suffer from Down syndrome with periodontal disease and peri-implant failure, compared to Down syndrome patients without periodontal disease and without peri-implant failure.

**Figure 5:**
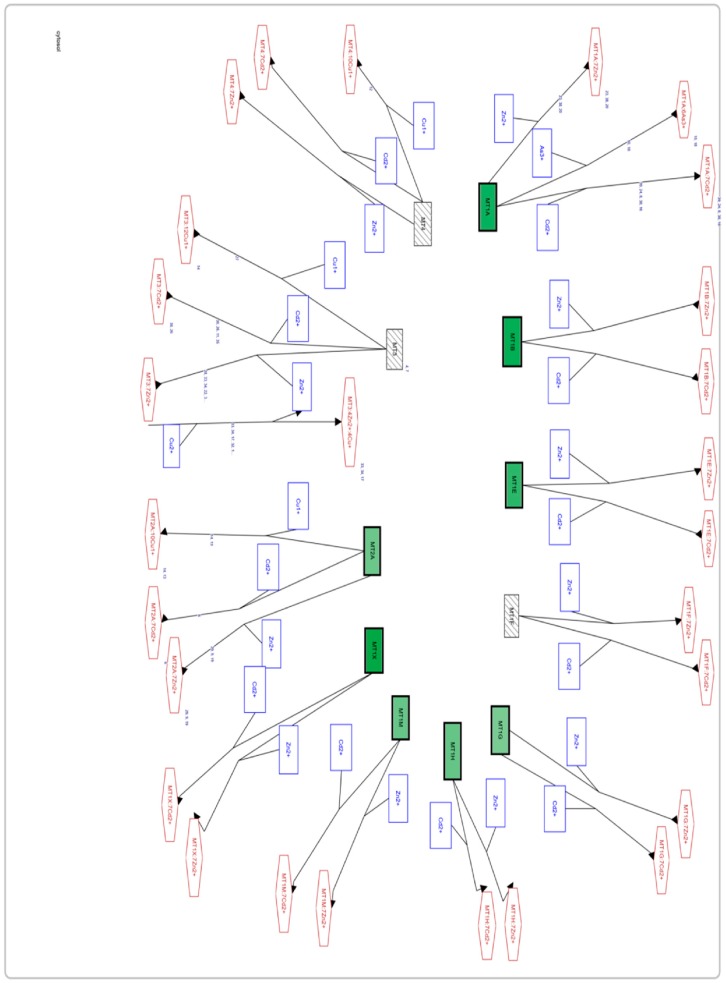
Screenshot of the Affymetrix Microarrays program on the metabolic pathway of the metallothioneins. Differences in genetic expression between the study groups are indicated, using the -PD-FI group as a control. Those genes that are downregulated are shown in green, upregulated genes (none are to be seen in the image) are in red, and genes with unaltered expression are in striped gray).

In our study results, genes corresponding to metallothioneins that are downregulated are only mainly MT1 and MT2A isoforms. However, for MT3 and MT4 no altered gene expression was found (Figure 5).

## 4. Discussion

The results of the study confirm the alternative hypothesis, that metallothioneins are expressed inconsistently. The metallothioneins are downregulated in Down syndrome patients with periodontal disease and failed implants.

The clinical question that forms the basis of the investigation is whether it is possible to identify any alteration of genetic expression that is responsible for the appearance of periodontitis and loss of dental implants in patients with Down syndrome. In this sense, the focus of our research is when both situations occur together (not mixed; it is a unique clinical situation when both circumstances occur), because it really is a problematic clinical situation. If there is no periodontitis, there will probably be no dental loss in this type of patient. If there is no rejection, implant therapy is valid. That is, the clinical problem appears when both circumstances occur, so it is the focus of the study.

Studying both situations separately would be interesting, to identify the factors involved in each of these situations. This could be a research project for a later time since it presents some difficulties in its realization: from the outset, two more groups would be necessary: one of patients with Down syndrome, with implant failure, and without periodontal disease. This group, at least in our clinical experience, does not exist (which means that it will be very scarce). A second necessary group is patients with Down syndrome, without periodontal disease and without implant rejection. Although we have identified a small number of patients who would fit into this group, their number and condition do not make their inclusion in this study viable for the moment.

On the other hand, and despite not knowing in detail all the activities that metallothioneins can perform, using the published bibliography about them, together with the results obtained, we can reach an important hypothesis about the involvement of MTs both in osseointegration of implants in Down syndrome patients and in the onset of periodontal disease in these patients.

Oxidative stress has been related to the inhibition of osteoblast cell differentiation. A study by Liu et al. [16] shows that hydrogen peroxide (H_2_O_2_) inhibits alkaline phosphatase activity in bone marrow stromal cells (BMSCs), among other early markers of bone-forming cell differentiation. To confirm the involvement of metallothioneins in this process, a culture of cells was attempted with prior MT treatment; it was observed that these protected the cells from inhibition by reactive oxygen species.

This study not only highlights the important role of metallothioneins as powerful antioxidant agents, which was already supported by numerous in vitro studies [17], but also how these have considerable significance when cell differentiation takes place in new bone formation.

The line of research from a previous study [16] continues to explore changes in expression in the different isoforms of MT in osteoporotic males and postmenopausal women. However, the researchers do not specify which metallothioneins were administered in the study cultures.

In this respect, in another study [18] where an assessment is made of whether metallothioneins play an important role in osteoblast differentiation and their level of expression in this process, the focus is only on MT1, MT2, and MT3 isoforms, also assessing the RNAm levels in alkaline phosphatase and osteocalcin to take them as reference points. In their results, no MT3 RNAm was detected at any time; with regard to MT1 and MT2, the RNAm levels rose significantly in the first 24 h in a culture treated with dexamethasone (Dex). This would show that MT1 and MT2 could develop a decisive role during the first stages of mesenchymal stromal stem cells (MSC) into osteoprogenitor bone cells. In fact, on adding an antisense oligonucleotide (a nucleotide chain with a sequence of bases complementary to a fragment of RNAm that would prevent its transcription) for MTs, it was observed that osteoblast differentiation and mineralization were considerably suppressed.

The fact that the expression of MT1 and MT2 genes seems to have important action during the first stages of bone-forming cell differentiation is an interesting starting point that strengthens the hypothesis put forward about the possible causes of failure of implants, and the lack of osseointegration, in Down syndrome patients. We cannot rule out that the condition evidenced by our study is related to the other pathological condition that differentiates both groups—that is, the onset of periodontal disease.

It is important to highlight that a low gene expression of MT1 and MT2 in Down syndrome patients in our study has been observed when comparing patients with periodontal disease and failed implant treatment with patients who do not suffer from periodontal disease and have a positive experience of implant treatment, but we do not know what expression metallothionein genes would have if another study group with different characteristics of periodontal disease and implant evolution were used.

Alterations in the expression of the MT1 and MT2 genes can lead to clinical alterations in patients with Down syndrome, such as severe periodontal disease, implant loss, or peri-implantitis; they may be related to the aforementioned role of these molecules in the early stages of bone healing, which can lead to a failure of osseointegration or to the presence of a bone with little strength to withstand the aggressions of the oral environment—this being especially important in the case of both periodontitis and peri-implantitis. This points to a possible line of research that could put the data provided in this study into perspective.

Conversely, we should not forget that these results need to be studied in greater depth, starting with the validation of the gene expression identified in this study, using a complementary technique, which would confirm the initial results. Subsequently, the study should focus on genes with confirmed altered expression. On the other hand, more efforts should also be made to increase the number of patients and obtain larger samples in each group, as well as complementary groups, so that the analyses can gain solidity.

## 5. Conclusions

With the limitations of our study, the low MT1 and MT2 gene expression seems to be related to the onset of periodontal disease and implant rejection in Down syndrome patients, although more data are required to confirm whether this relationship is due to one of the two conditions, to both independently, or to the two jointly—this last option being indicated by our current study.

## Figures and Tables

**Table 1:** Clinical data of the patients studied. Patients 9‒11 did not have implants; it was assumed, from our clinical experience, that in the absence of periodontal disease, they would not be candidates for early failure after implant placement, in order to increase the number of patients in the control group. In any case, the results of the analysis, eliminating these patients, were similar.

Patient	Group	Age	Sex	Smoker	Drinker	History of Controlled Periodontal Disease	Implants Placed	Bone Regeneration	Result after Two Years of Follow-up
1	+PD+FI	41	Female	No	No	Yes	2 implants	No	1 implant lost and 1 implants with severe peri-implantitis
2	+PD+FI	39	Female	No	No	Yes	3 implants	No	1 implant lost and 2 implants with severe peri-implantitis
3	+PD+FI	33	Male	No	No	Yes	4 implants	No	2 implants with severe peri-implantitis
4	+PD+FI	35	Male	No	No	Yes	12 implants	No	3 implants lost
5	-PD-FI	40	Female	No	No	No	3 implants	No	No implant failure or peri-implantitis
6	-PD-FI	34	Female	No	No	No	2 implants	No	No implant failure or peri-implantitis
7	-PD-FI	43	Female	No	No	No	3 implants	No	No implant failure or peri-implantitis
8	-PD-FI	48	Female	No	No	No	2 implants	No	No implant failure or peri-implantitis
9	-PD-FI	44	Male	No	No	No	No	No	No implant failure or peri-implantitis
10	-PD-FI	38	Male	No	No	No	No	No	No implant failure or peri-implantitis
11	-PD-FI	44	Male	No	No	No	No	No	No implant failure or peri-implantitis

**Table 2:** Results table, with significant values, using Transcriptome Analysis Console Software 4.0 (ThermoFisher Scientific, Waltham, MA, USA) and 4.0.1.

ID	+PD+FI (Average, log 2)	-PD-FI (Average, log 2)	Fold Change	*p* Value	Fdr *p* Value	Genetic Symbol	Description
TC1600007959	8.05	9.49	−2.71	0.0014	0.9997	MT1E	Metallothionein 1E
TC1600007966	6.8	8.06	−2.39	0.0018	0.9997	MT1H	Metallothionein 1H
TC1600011399	10.73	12.36	−3.09	0.0021	0.9997	MT1X	Metallothionein 1X
TC1600007962	12.17	13.67	−2.82	0.0023	0.9997	MT1A	Metallothionein 1A
TC1600007964	11.7	13.38	−2.95	0.0024	0.9997	MT1B; MT1C	Metallothionein 1B; Metallothionein 1C, pseudogene
TC1600007958	11.58	12.93	−2.55	0.0048	0.9997	MT1L	Metallothionein 1L (gene/pseudogene)
TC1600007957	13.82	15.05	−2.35	0.0072	0.9997	MT2A	Metallothionein 2A
TC1600007960	9.03	10.39	−2.37	0.0092	0.9997	MT1M	Metallothionein 1M
TC1600010421	10.62	11.79	−2.24	0.0118	0.9997	MT1G	Metallothionein 1G

Filter values of *p*-value have been correctly established as 0.05/Filter values of Fold Change have been established between 2 and −2./The SW TAC will use the correct version of R for the analysis/Fdr = False discovery rate.
